# P-1738. Immune Reconstitution Inflammatory Syndrome in HIV/AIDS: A Challenging Case of Co-Infections with Coccidioidomycosis and Cytomegalovirus

**DOI:** 10.1093/ofid/ofaf695.1909

**Published:** 2026-01-11

**Authors:** Kaushal Patel, Vedant Shah, rosemaria Cyriac Nirappel, Glen I Abdo

**Affiliations:** New York Medical College, Parsipanny, New Jersey; New York Medical College, Parsipanny, New Jersey; New York Medical College, Parsipanny, New Jersey; NYMC at St. Mary's General Hospital and Saint Claire's Health, Elmhurst, New York

## Abstract

**Background:**

Coccidioidomycosis, or Valley Fever, is a fungal infection endemic to the southwestern United States and certain regions of Central and South America. Although often a self-limiting respiratory condition, widespread disease may manifest in immunocompromised individuals, especially those with HIV/AIDS. The implementation of antiretroviral medication (ART) has markedly diminished opportunistic infections; nevertheless, it also presents the danger of Immune Reconstitution Inflammatory Syndrome (IRIS), which may paradoxically exacerbate pre-existing infections or reveal subclinical infections.

**Figure:**
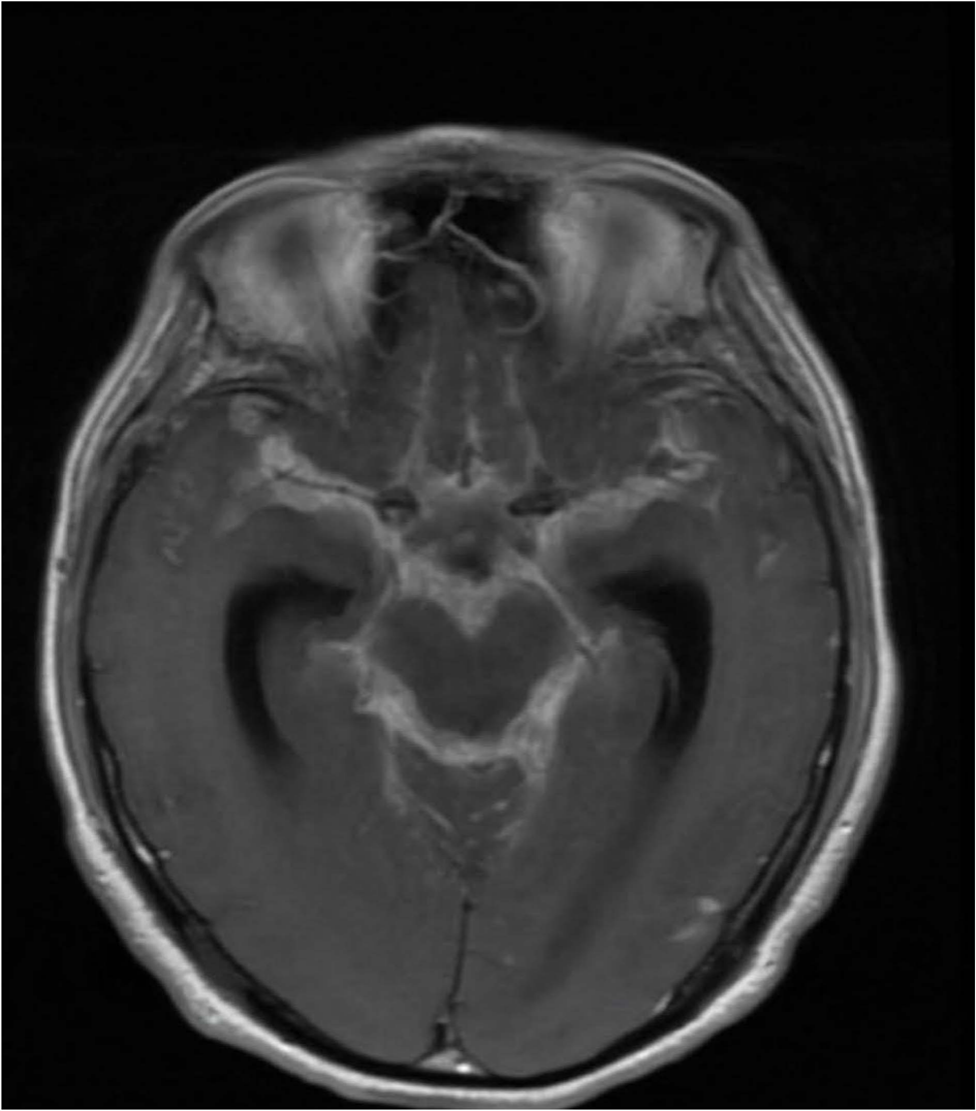
Meningitis Diffuse sulcal, basal cistern, posterior fossa subarachnoid space enhancement consistent with diagnosis of meningitis

**Figure:**
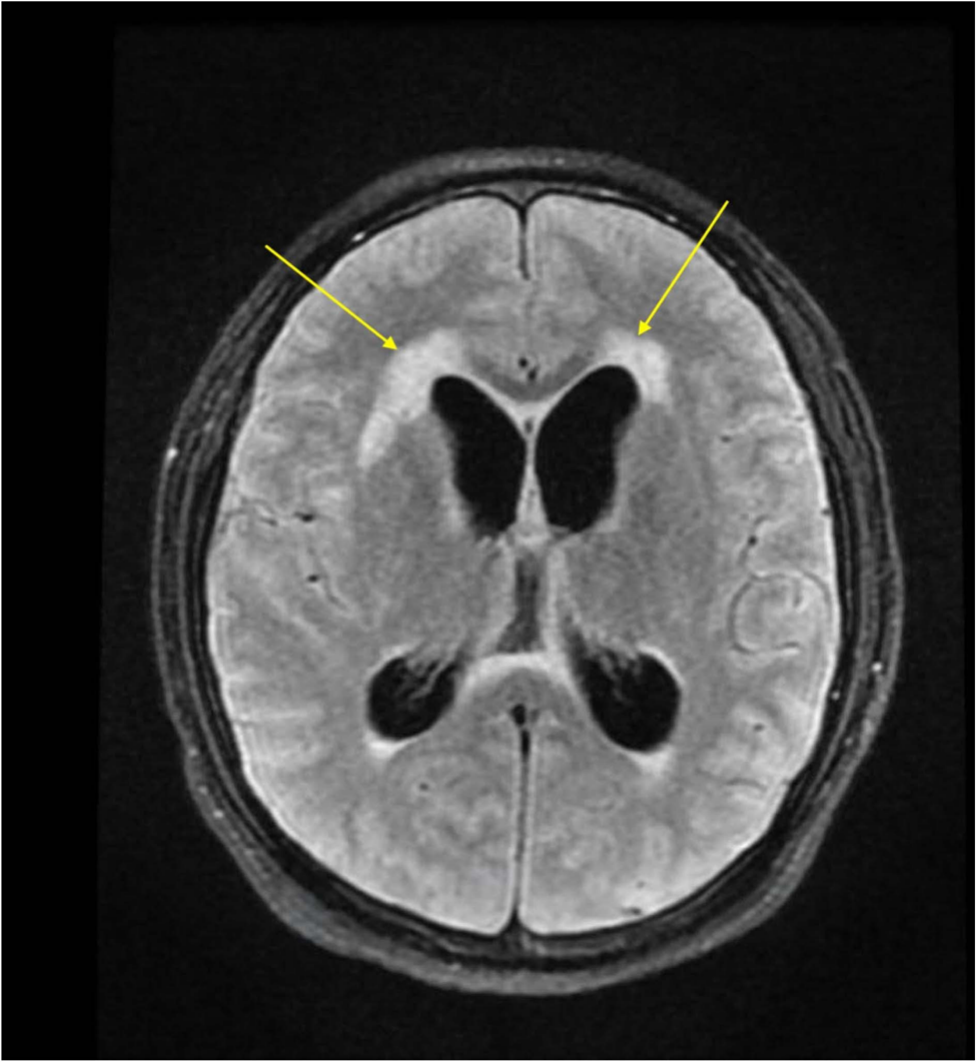
Fungal infection Transependymal CSF absorption which suggest fungal infection

**Methods:**

We present a case of a 32-year-old HIV-positive male who experienced IRIS following the commencement of ART in the context of widespread coccidioidomycosis. The patient initially exhibited gradual weight loss, respiratory symptoms, and gastrointestinal hemorrhage, resulting in a diagnosis of disseminated coccidioidomycosis, esophageal candidiasis, and HIV/AIDS with a CD4 count of less than 40 cells/µL. Notwithstanding the commencement of ART and antifungal treatment, the patient later manifested CMV meningitis, coccidioidal meningitis accompanied by hydrocephalus, and paradoxical IRIS, resulting in numerous hospitalizations.

**Figure:**
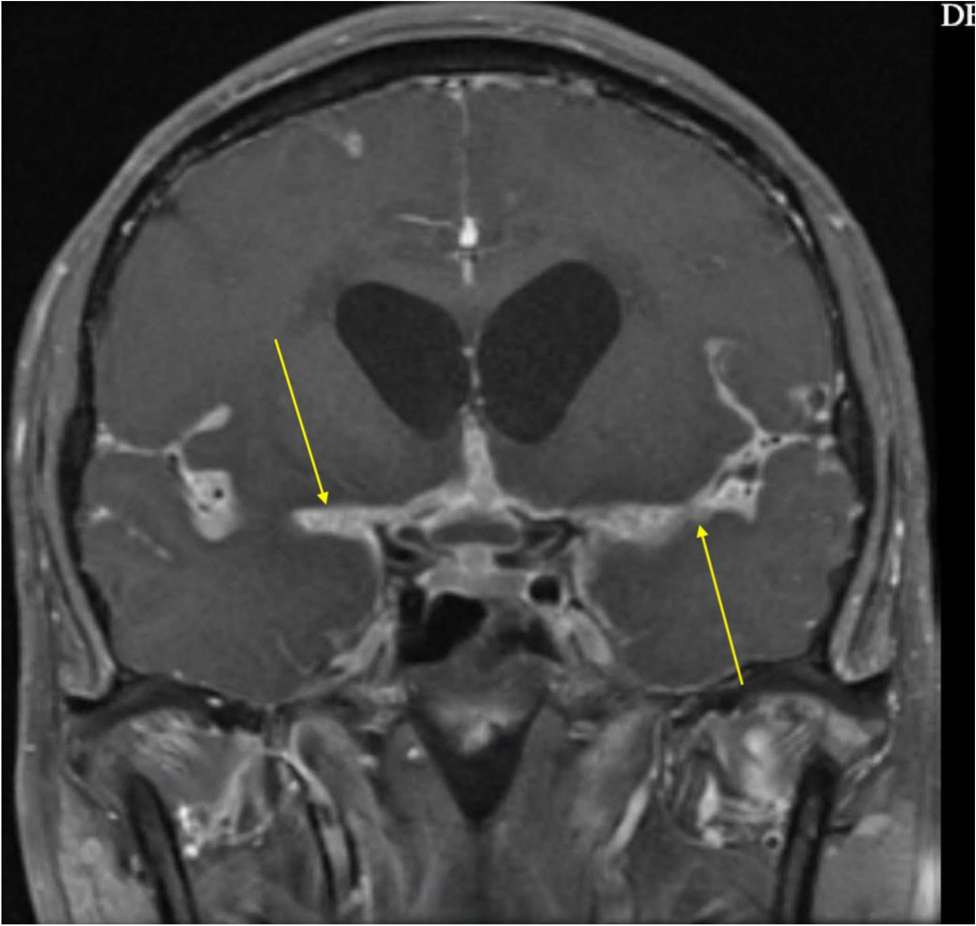
Image shows white enhanced areas indicating CSF that is usually black

**Results:**

Management comprised high-dose fluconazole, ganciclovir, repeated lumbar punctures for symptomatic alleviation of intracranial hypertension, and the continuation of antiretroviral therapy. The patient's CD4 level gradually enhanced, and his symptoms became stable.

**Conclusion:**

This case highlights the challenges of managing IRIS in disseminated coccidioidomycosis with CNS involvement and opportunistic infections. The paradoxical worsening of symptoms required careful balancing of immune recovery and infection control with prolonged antifungal therapy and intracranial pressure management. Unlike mycobacterial or cryptococcal IRIS, corticosteroid use in fungal IRIS remains unclear, complicating treatment decisions.

Additionally, as climate change expands the endemic range of Coccidioides, increasing awareness of coccidioidomycosis and its complications is essential. Early recognition, adherence to ART, and a multidisciplinary approach are crucial for improving outcomes in these complex cases.

**Disclosures:**

All Authors: No reported disclosures

